# Multiuser Application for the Diagnosis and Treatment of Depression in Women’s Self-Help Groups: Pilot Randomized Controlled Trial

**DOI:** 10.2196/68052

**Published:** 2025-10-01

**Authors:** Amritha Bhat, Ruben Johnson-Pradeep, Bharat Kalidindi, Dhinagaran Devadass, B Ramakrishna Goud, Tony Raj, Sumithra Selvam, Yesenia Navarro-Aguirre, Pamela Y Collins, Krishnamachari Srinivasan

**Affiliations:** 1 Department of Psychiatry and Behavioral Sciences, University of Washington, 1959 NE Pacific Street, Box 356560, Seattle, WA, 98195, United States, 1 2065433117; 2 Department of Psychiatry, St John’s Medical College, Bengaluru, India; 3 Division of Mental Health & Neurosciences, St. John's Research Institute, Bengaluru, India; 4 Division of Medical Informatics, St. John's Research Institute, Bengaluru, India; 5 Department of Community Medicine, St John’s Medical College, Bengaluru, India; 6 Department of Physiology, St. John's Medical College, Bengaluru, India; 7 Division of Epidemiology, Biostatistics and Population health, St. John's Research Institute, Bengaluru, India; 8 Department of Anthropology, University of Washington, Seattle, WA, United States; 9 Department of Mental Health, Johns Hopkins Bloomberg School of Public Health, Baltimore, MD, United States; 10 Department of Psychiatry & Behavioral Sciences, The Johns Hopkins University School of Medicine, Baltimore, MD, United States

**Keywords:** women, depression, community-based, mobile mental health, rural

## Abstract

**Background:**

Depression in women results in elevated morbidity rates, functional impairment, diminished quality of life, and an increased risk of suicide. Numerous obstacles impede access to mental health treatment for women in India. Digital mental health solutions can bridge the treatment gap, but it is important to tailor these solutions to the context and to end-users.

**Objective:**

We conducted a pilot randomized controlled trial to test the feasibility, acceptability, and preliminary effectiveness of a mental health app deployed in community-based organizations in improving depression outcomes.

**Methods:**

The Multiuser Interactive Health Response Application (MITHRA) is a multiple-user mobile app used in community-based organizations for screening, tracking, and supporting stepped-care treatment for depression. MITHRA is based on the healthy activity program, a brief psychological intervention based on behavioral activation. It includes audio, video, and enhanced touchscreen capabilities to overcome the barrier of illiteracy and lack of access. It was developed in collaboration with a participatory design group consisting of primary and secondary end-users and is available on tablets installed in self-help groups (SHGs), which are community-based organizations in India. The SHGs were randomized to MITHRA (n=3) or enhanced usual care (EUC; n=3). During SHG meetings, women completed the Patient Health Questionnaire-9 (PHQ-9). Based on their PHQ-9 scores, they were assigned different modules. In the EUC SHGs, women viewed one module of education on symptoms of depression. Primary outcomes include feasibility and acceptability, and secondary outcomes include depressive symptoms and functioning. Repeated-measures ANOVA was performed to compare the change in the outcome scores over time between study groups. A *P* value of<.05 was considered statistically significant.

**Results:**

MITHRA was found to be feasible and acceptable. A total of 96% of intervention arm participants completed at least half of their assigned modules. Although not powered for effectiveness outcomes, in this trial, we found that the change at 6 months from baseline in depressive symptoms (PHQ-9) were significantly different between MITHRA and EUC (*P*=.037), with greater improvement in the intervention group. Similarly, significant improvement in the World Health Organization Disability Assessment Scale score was noted in the MITHRA group (*P*=.005).

**Conclusions:**

MITHRA is feasible and acceptable for use in women’s SHGs. Larger studies should examine the effectiveness of this approach in identifying and treating depression.

## Introduction

Mental disorders are among the top 10 leading causes of global disease burden [1]. Depression is the most common mental disorder and is twice as common in women than in men [2]. Women in general experience several sources of stress such as being the sole childrearing adult in a household, managing multiple roles, unequal power relations with men, a sense of powerlessness [3], and higher rates of poverty [4], all of which are associated with a higher risk of depression.

Women with depression, even of mild to moderate severity, experience high rates of morbidity, functional impairment, low quality of life [5], and higher risk of suicide [6]. The effects of depression in women impact families as well given the multidimensional roles that they play [7]. Yet, depression often goes undiagnosed and untreated in women in India due to stigma, childcare, gender inequality, and mental health workforce deficits, impacting mental health treatment access [8]. Only one woman attends an outpatient mental health appointment for every three men in India [8], and untreated depression among women is a major public health problem. While digital mental health solutions can help close this treatment gap, there is a need to adapt these digital mental health solutions to the context and to specific users. For example, while app-based depression screening and treatments are widely used and effective, issues such as shared ownership of phones, which is very common in India [9], may pose a barrier to the widespread use of digital mental health solutions. In general, access to mobile phones is lower among women in India, especially in rural India, as women have time allocation constraints on phone usage, limited digital skills, and often depend on men for phone ownership [10,11].

Given these contextual considerations, we used a participatory design to develop and test an app—Multiuser Interactive Health Response Application (MITHRA)—that screens for depression and provides a brief behavioral intervention [12]. This is a multiuser app deployed in self-help groups (SHGs), which are community-based organizations for women. The conceptual model for MITHRA is based on Fortney et al’s [13] access to care model, and MITHRA targets actual and perceived barriers to access identified by patients. MITHRA uses nonencounter-based screening, tracking, and low-intensity interventions, addressing the barriers of stigma and travel time. MITHRA addresses the perceived need for care among women by providing education about depression. The process of app development has been described previously [14]. Briefly, women are presented with multimedia modules to view based on their Patient Health Questionnaire-9 (PHQ-9) score. Women who score <5 receive a prompt to watch general information on depression; women who score 5 or higher watch short (10 to 15 min) interactive multimedia-based modules based on the Healthy Activity Program [15], a brief behavioral intervention.

Here, we describe a pilot randomized controlled trial (RCT) designed to test the feasibility, acceptability, and preliminary effectiveness of MITHRA in improving depression outcomes.

## Methods

### Ethical Considerations

All study activities were approved by the Institutional Review Board at the University of Washington (no. STUDY00010415, approved 8 June 2020), and the Institutional Ethics Committee at St. John’s Medical College (IEC Study Ref. no. 184/2020, approved 24 July 2020). We obtained informed consent from all participants; for those who could not read, we explained the study details and obtained consent by thumbprint and by recording verbal consent, witnessed, and signed by a neutral observer. Data for analyses were deidentified. Participants received INR 200 (US $ 2.50) for each assessment/interview

### Study Sites

The study site was within Anekal taluk (subdistrict administrative block of a state in India) of Karnataka, which is approximately 25 miles from Bengaluru. We included 10 villages with functional SHGs with a population of 9724 people [9]. Focus groups, usability testing, and deployment of the tablet-based apps for the pilot RCT were all conducted in these SHGs. The methods for the pilot RCT are described below.

### Randomization and Masking

We randomized three SHGs to use MITHRA and three SHGs to enhanced usual care (EUC). A simple randomization list was generated, and the eligible SHGs were allocated into MITHRA or EUC. Participating SHGs were in different villages, with no possibility of contamination or exchange of information about the intervention. Study investigators were blinded to the random allocation. At recruitment, 3 months, and 6 months, a research assistant administered outcome assessments over the telephone to avoid unmasking of the SHG randomization status (presence of tablets in the SHGs). The research assistant only had access to the name and mobile number of the participant, and no details on the participant’s village of residence or SHG.

### Intervention

MITHRA was available on tablets placed in an assigned private place in each SHG randomized to MITHRA. Women typically attend SHG meetings two to three times a month, and the use of MITHRA at every attendance was encouraged. They logged into MITHRA with a fingerprint secure single-user sign-on to complete depression screening using the Patient Health Questionnaire-9 (PHQ-9). On completion of the PHQ-9, each woman received a prompt based on her total score and viewed the recommended modules. Users could unlock virtual reward points/badges on completion of the required questionnaires, modules, and activities. Any woman who scored anything other than 0 on question 9 of the PHQ-9 (ie, the suicidal ideation item) would be prompted to call the community health worker associated with that SHG. For these women, the app would also trigger an alert to the community health worker who would immediately contact the SHG administrator on site and call the patient to complete a suicide risk assessment [16]. Community health workers were trained in safety protocols.

In the EUC SHGs, women watched a module offering education regarding the symptoms of depression.

### Data Analysis

Intent-to-treat analyses were performed. We used the Q-Q plot to test assumptions of normality. We used descriptive statistics to assess our primary outcome, the app usage rates. We used the mean and SD for normally distributed variables measured at baseline, 3 months, and 6 months. Variables measured included depressive symptoms measured on the Quick Inventory of Depressive Symptoms (QIDS) scale [17], functioning symptoms on the World Health Organization Disability Assessment Scale (WHODAS) [10], and behavioral activation on the Behavioral Activation Depression Scale (BADS) [11]. We used the *χ*^2^ test or Fisher exact tests, as appropriate, to compare the categorical variables at baseline between the study groups. At each time of assessment, we compared the outcome parameters between study groups using independent *t* tests or Mann-Whitney *U* tests, as appropriate. The change in outcomes at 3 and 6 months from baseline was compared between the study groups using the Mann-Whitney *U* test. Within each study group, the McNemar *χ*^2^ test was used to compare the change in the proportion of depression categories (minimal, and mild to moderate) from baseline. In addition, repeated-measures ANOVA was performed on log-transformed outcome variables to compare the change in the outcome scores over time between the study groups. A *P* value of <.05 was considered statistically significant. All analyses were performed using SPSS (version 26.0; IBM Corp.).

## Results

**Figure 1.**
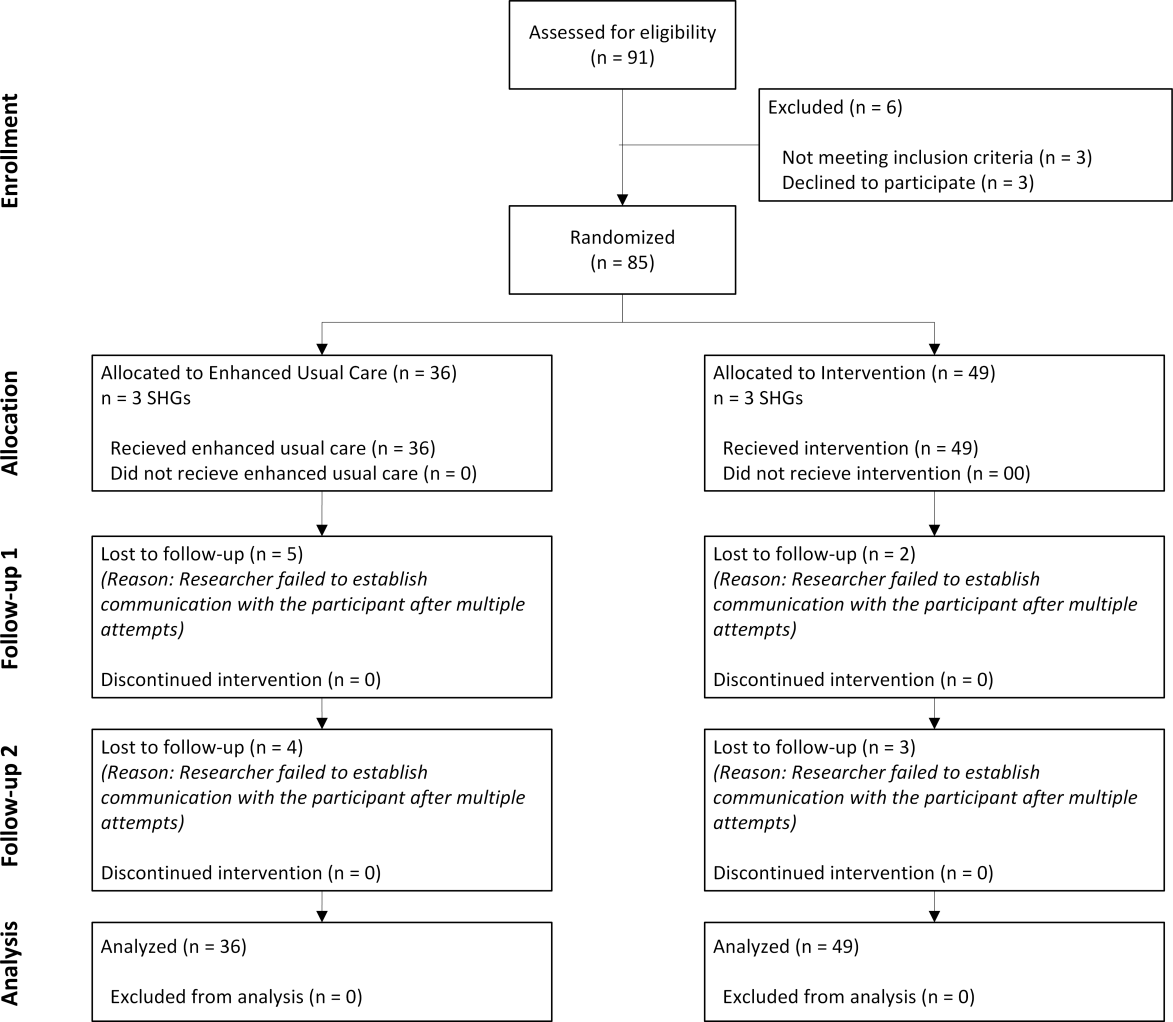
Enrollment flow chart. SHGs: self-help groups.

The flowchart showing participant inclusion is shown in Figure 1.

### Participant Characteristics

The baseline characteristics of the study participants are provided in Table 1 and were comparable between study groups except for the type of family, with a significantly higher proportion of participants belonging to nuclear families in the intervention group compared to the EUC group. At baseline, the PHQ-9 and QIDS scale scores were significantly higher and the WHODAS score was significantly lower in the intervention group than in the EUC group.

**Table 1.** Baseline characteristics of participants.

Variable	MITHRA group (n=49)	EUC group (n=36)
Age, y; mean (SD); range, 18-63 y	41.6 (10.5)	40.7 (11.9)
Marital status		
Married	49 (100)	33 (91.7)
Never married/Widow	0	3 (8.3)
Education, n (%)		
No schooling	23 (46.9)	19 (52.8)
Primary–Middle school	11 (22.5)	2 (5.5)
Secondary-Higher Secondary	12 (24.5)	11 (30.6)
Graduation/Diploma	3 (6.1)	4 (11.1)
Type of family, n (%)^a^		
Nuclear	41 (83.7)	19 (52.8)
Joint	8 (16.3)	17 (47.2)
Occupation, n (%)		
Unskilled manual work	27 (55.1)	20 (55.6)
Skilled manual work	10 (20.4)	14 (38.9)
Homemaker	12 (24.5)	1 (2.8)
Working professional	0	1 (2.8)
Monthly family income, n (%)		
≤INR 10,000 (US $112.65)	22 (44.9)	10 (27.8)
> INR 10,000 (US $112.65)	27 (55.1)	26 (72.2)
Years of association with self-help groups, n (%)		
<5 years	22 (44.9)	10 (27.8)
5-10 years	9 (18.4)	10 (27.8)
>10 years	18 (36.7)	16 (44.4)
Baseline measures, mean (SD)		
PHQ-9^a^^b^	4.24 (3.35)	3.03 (3.40)
QIDS scale^a^^c^	4.18 (2.78)	3.25 (3.36)
WHODAS^a^^d^	27.78 (4.73)	29.92 (3.64)

a P<.05

b PHQ-9: Patient Health Questionnaire-9.

c QIDS: Quick Inventory of Depressive Symptoms.

d WHODAS: World Health Organization Disability Assessment Scale.

### Feasibility

#### Feasibility of Randomization and Masking

Randomization was feasible, although some participant level characteristics were significantly different between the intervention and EUC groups. Patient data continued to be masked, as the research assistant was not aware of group assignment during the phone-based research assessments.

#### Retention

At the 3-month follow-up, we had 47/49 and 31/36 complete assessments for the intervention and EUC groups, respectively. At the 6-month follow-up, we had 46/49 and 32/36 complete assessments for the intervention and EUC groups, respectively, with retention rates of 92% at both 3 and 6 months.

#### Safety

There were no adverse events, and no participants reported suicidal ideation or required a referral to the Primary Health Center per the protocol.

### App Usage Rates

Only the MITHRA arm was considered, and completion of 50% of the modules was considered adequate usage. Of the 49 women assigned to MITHRA, 47 completed 50% of the modules.

### Effectiveness of the Intervention

The change in outcome parameters at 3 and 6 months from baseline between the MITHRA and EUC groups is reported in Table 2. At 6 months, the change in the PHQ-9 score from baseline was significantly different between the study groups (*P*=.037), with greater improvement in the intervention group. Although statistically non-significant, the change in the QIDS scale score was greater in the intervention group than in the EUC group (*P*=.07). There was no significant difference in the BADS score at 6 months. The change in the WHODAS score was significantly different between the study groups (*P*=.005), with more improvement in the intervention group.

**Table 2.** Comparison of outcome parameters between study groups at baseline, 3, and 6 months.

Outcome parameter	MITHRA group (n=49)	EUC group (n=36)	*P* value^a^
PHQ–9^b^ score, median (IQR)			
Baseline	4 (1 to 6)	2 (0.25 to 4.75)	.04
Change at 3 months	-2 (-4 to ‐1)	-1 (-4 to 0)	.20
Change at 6 month	-3 (-5 to ‐0.75)	-1 (-3 to 0)	.04
QIDS scale^c^ score, median (IQR)			
Baseline	4 (2 to 5)	2.5 (0 to 4)	.04
Change at 3 months	0 (-2.0 to 1.0)	0 (-2.0 to 1.0)	.58
Change at 6 months	-1.0 (-3.0 to 1.0)	0 (-0.75 to 2.75)	.07
BADS^d^ score, median (IQR)			
Baseline	30.0 (27.0 to 33.5)	34.0 (28.0 to 38.7)	.13
Change at 3 months	1 (-3.0 to 5.0)	0 (-4.0 to 3.0)	.33
Change at 6 months	1.50 (-3.0 to 6.0)	0 (-5.0 to 4.0)	.19
WHODAS^e^ score, median (IQR)			
Baseline	27.0 (24.5 to 31.0)	29.5 (27.0 to 33.0)	.03
Change at 3 months	-1.0 (-4.0 to 1.0)	-1.0 (-4.0 to 0)	.48
Change at 6 months	0.50 (-3.0 to 4.0)	-3.0 (-5.0 to 0)	.005

a *P* values were obtained using the Mann-Whitney *U* test at each time point.

b PHQ-9: Patient Health Questionnaire-9.

c QIDS: Quick Inventory of Depressive Symptoms.

d BADS: Behavioral Activation Depression Scale.

e WHODAS: World Health Organization Disability Assessment Scale.

The PHQ-9 scores were categorized as minimal and mild-to-moderate and compared by study groups, as presented in Table 3. There was no significant difference in the proportion of participants with mild-to-moderate depression between the study groups. However, within-group analysis showed a significant reduction in the proportion of participants with mild-to-moderate depression from baseline to 6 months in the intervention group (*P*<.001).

**Table 3.** Comparison of Patient Health Questionnaire-9 (PHQ-9) scores between the study groups.

PHQ-9 score	MITHRA group (n=49)	EUC group (n=36)	*P* value
PHQ-9 score at baseline^a^, mean (SD)			.09
Minimal depression	28 (57.1)	27 (75.0)	
Mild-to-moderate depression	21 (42.9)	9 (25.0)	
PHQ-9 score at 3 months, mean (SD)			.76
Minimal depression	46 (97.9)	30 (96.8)	
Mild-to-moderate depression	1 (2.1)	1 (3.2)	
PHQ-9 score at 6 months, mean (SD)			.19
Minimal depression	42 (91.3)	26 (81.3)	
Mild-to-moderate depression	4 (8.7)^b^	6 (18.8)	

a For within-group comparisons, the change from baseline to that at 6 months was calculated using the McNemar chi-square test.

b *P*<.001

The results of the additional analysis using repeated-measures ANOVA revealed that there was a significant time effect for the PHQ-9, BADS, and QIDS scale scores, indicating that the change in the outcome scores over time was statistically significant (p<0.01). However, there was no significant interaction between time and the study groups for any of the outcomes.

## Discussion

This study demonstrates the feasibility and acceptability of deploying a culturally tailored app-based depression intervention (MITHRA) within women’s SHGs in rural India. MITHRA is designed to address the large treatment gap for depression in women. It was developed in consultation with key end-users, it is culturally appropriate (settings and characters in the video reflect the users), available in the local language, and based on an evidence-based intervention.

We successfully completed a pilot cluster RCT comparing MITHRA to EUC to assess the feasibility of recruitment and randomization, acceptability, and preliminary effectiveness. In this RCT, we randomized participants to app-based depression screening and treatment in community-based settings. Participants in the control arm received EUC; they viewed one module of education on the symptoms of depression and how to seek help. Typical or usual care in SHGs includes no screening or education regarding depression. We found that it is feasible to recruit and randomize SHGs to app-based treatment or usual care, and to obtain consent from SHG members to participate in a clinical trial. Our pilot cluster RCT adds to a small but growing body of evidence supporting the use of digital mental health interventions for women in low-resource settings, particularly in South Asia, where access to mental health care remains limited. For example, a similar study in a low- or middle-income country highlights the high acceptability of mobile-based interventions among women with limited access to traditional mental health services [18].

Randomization was not balanced for patient-level characteristics such as socioeconomic status and depression, a limitation that can occur in cluster RCTs [19]. We controlled for these characteristics in our analysis. We had an excellent retention rate of 92% at both the time-points of 3 months and 6 months. Women in the intervention arm engaged well with the app, with 96% of the women completing at least 50% of the modules assigned to them. This suggests that carefully designed locally contextualized digital tools can be both feasible and acceptable in these settings. We also demonstrated the feasibility of collecting the required data and conducting analyses.

Preliminary analyses showed that symptom scores as measured by the PHQ-9 and QIDS scales decreased over time in women assigned to MITHRA compared to EUC. Our post-hoc analysis showed that in MITHRA SHGs, the proportion of women with mild-to-moderate depression symptoms as measured using the PHQ-9 was significantly lower at 6 months compared to that at baseline. This proportion did not reach statistical significance in the EUC arm. While the study was not powered to detect efficacy, these signals are promising and aligned with broader evidence, indicating that even lower-intensity digital psychological interventions can lead to meaningful improvements in mental health outcomes.

Our findings also contribute to the growing literature on stepped care approaches for depression in low- or middle-income countries. MITHRA incorporates the core principles of stepped care by providing low-intensity scalable interventions for women with mild-to-moderate symptoms while also including protocols for referral or treatment intensification for those with severe symptoms. Although no women in our study met the criteria for severe depression or required stepped-up care, the infrastructure we established for monitoring and referral are aligned with best practices to allocate resources efficiently while ensuring that individuals receive appropriate levels of care. The absence of referrals in our sample may reflect the fact that women with more severe depressive symptoms are less likely to participate in SHGs, an important gap that future work must address. Similarly, no women in the study endorsed suicidal ideation; however, we did have appropriate treatment intensification protocols in place as part of the stepped care.

The limitations of our study include that our results may not be applicable to women with severe depressive symptoms who may not be attending SHG meetings. Additionally, as a feasibility study, our primary objective was to assess feasibility and acceptability, and we were not powered to assess effectiveness. The preliminary data generated will inform the design of a fully-powered RCT. In addition, the study was funded in 2020, and India was in a national lockdown for 2 months in early 2020 due to the COVID-19 pandemic. This delayed the study onset, as community gatherings were not allowed.

We found that it is feasible to conduct a pilot RCT of an app-based depression screening and brief behavioral intervention in community-based organizations in rural India. App-based depression screening and treatment such as that offered by MITHRA can help address barriers to mental health treatment access such as transportation and mental health workforce deficits. Deploying MITHRA in women’s SHGs can also help address gender-specific stigma and access to treatment. Widespread implementation of community-based solutions such as MITHRA that focus on the prevention of depression onset or treatment of mild-to-moderate symptoms should always be coupled with a plan to intensify care as needed for those with severe symptoms. The next steps are to test the effectiveness of this approach in the treatment of depression in women in a larger RCT.

## Supplementary material

10.2196/68052 Checklist 1 CONSORT checklist.

## Abbreviations

BADS: Behavioral Activation Depression Scale
EUC: enhanced usual care
MITHRA: Multiuser Interactive Health Response Application
PHQ-9: Patient Health Questionnaire-9
QIDS: Quick Inventory of Depressive Symptoms
RCT: randomized controlled trial
SHG: self-help group
WHODAS: World Health Organization Disability Assessment Scale
